# A prenatal diagnosis case of partial duplication 21q21.1-q21.2 with normal phenotype maternally inherited

**DOI:** 10.1186/s12920-021-01013-x

**Published:** 2021-06-19

**Authors:** Chunyan Jin, Zhiping Gu, Xiaohan Jiang, Pei Yu, Tianhui Xu

**Affiliations:** Department of Medical Genetics and Prenatal Diagnosis, Hospital Affiliated 5 to Nantong University (Taizhou People’s Hospital), 399 Hailing South Road, Taizhou, 225300 Jiangsu China

**Keywords:** Down syndrome, Down syndrome critical region (DSCR), Partial duplication 21, Prenatal diagnosis

## Abstract

**Background:**

Down syndrome is characterized by trisomy 21 or partial duplication of chromosome 21. Extensive studies have focused on the identification of the Down Syndrome Critical Region (DSCR). We aim to provide evidence that duplication of 21q21.1-q21.2 should not be included in the DSCR and it has no clinical consequences on the phenotype.

**Case presentation:**

Because serological screening was not performed at the appropriate gestational age, noninvasive prenatal testing (NIPT) analysis was performed for a pregnant woman with normal prenatal examinations at 22 weeks of gestation. The NIPT results revealed a 5.8 Mb maternally inherited duplication of 21q21.1-q21.2. To assess whether the fetus also carried this duplication, ultrasound-guided amniocentesis was conducted, and the result of chromosomal microarray analysis (CMA) with amniotic fluid showed a 6.7 Mb duplication of 21q21.1-q21.2 (ranging from position 18,981,715 to 25,707,009). This partial duplication of 21q21.1-q21.2 in the fetus was maternally inherited. After genetic counseling, the pregnant woman and her family decided to continue the pregnancy.

**Conclusion:**

Our case clearly indicates that 21q21.1-q21.2 duplication is not included in the DSCR and most likely has no clinical consequences on phenotype.

## Background

Duplication or partial duplication of chromosome 21 leads to severe clinical phenotypes, including Down syndrome (DS), congenital heart defects, hypotonia, developmental delay, and speech delay, among others [1–3]. To delineate the genomic regions associated with a specific DS phenotype, several research groups have studied genotype–phenotype correlations by mapping partial duplication of 21 in cases with the DS phenotype, and the concept of DSCR is constantly being improved [4–6]. However, there are few cutting-edge analyses revealing the region of partial duplication of chromosome 21 that would not lead to the DS phenotype. This is mainly due to the scarcity of cases. Therefore, global cooperation is needed to report an increasing number of nonpathogenic cases of chromosome 21 partial duplication to provide a clinical basis for the systematic study of genotype–phenotype correlations of chromosome 21 with a normal phenotype. Prenatal diagnosis of a trisomy 21 patient without the DS phenotype revealed a derivative chromosome 21 that did not include the DSCR region [7]. Partial duplication of chromosome 21 from the centromere to band 21q21.3 results in a lack of the DS phenotype, though some clinical manifestations, such as "sandal gaps", joint hyperlaxity, hypotonia and brachycephaly, are present [8]. In the present case, a family was reported to carry the 21q21.1-q21.2 duplication, with no clinical phenotype. We hope this research provides benign clinical evidence of partial duplication of chromosome 21 for prenatal diagnosis and reduces the anxiety caused by the clinical uncertainty conveyed by chromosomal data analysts and genetic consultants.

## Case presentation

**Fig. 1:**
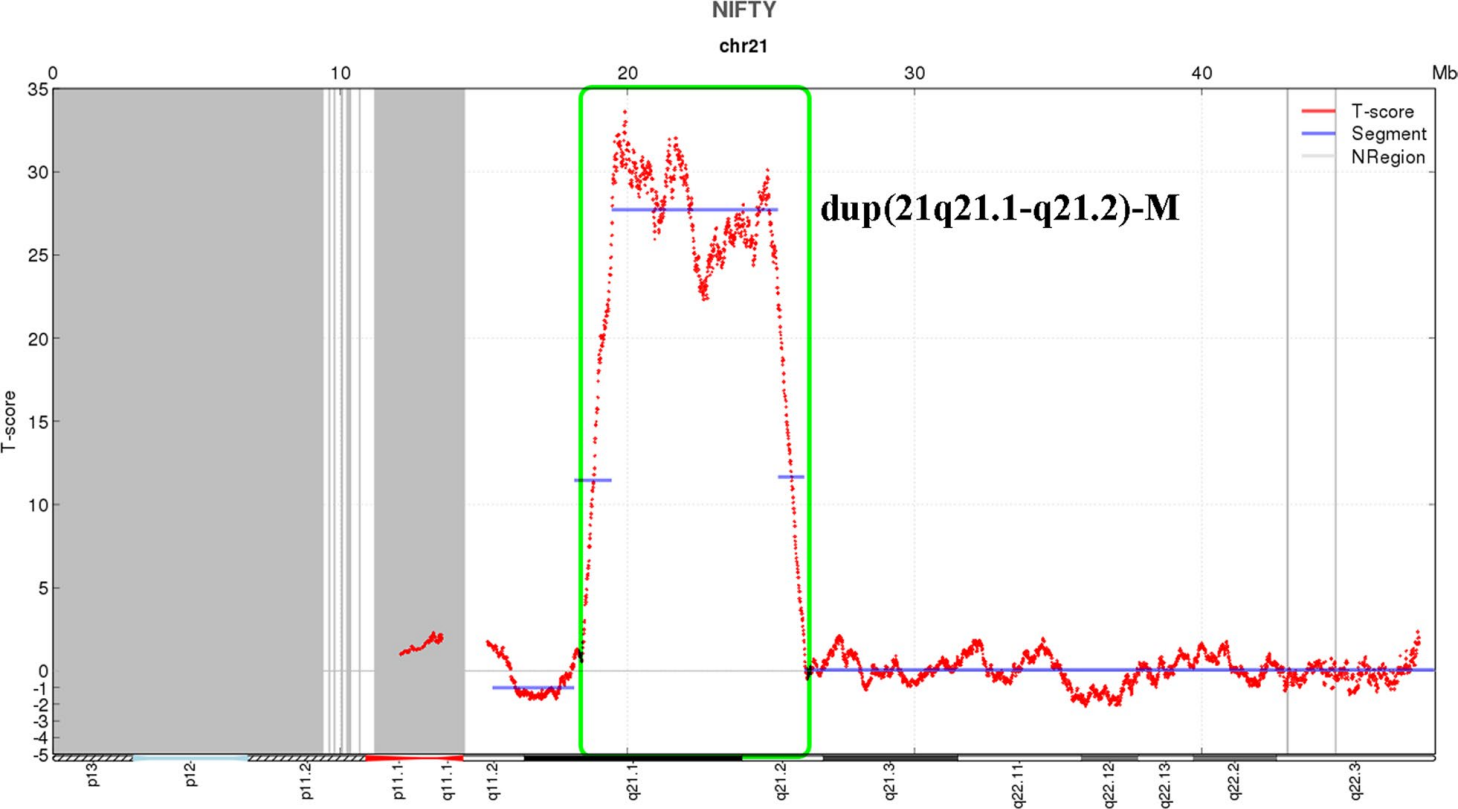
NIPT result showed a 5.8 Mb maternal duplication of 21q21.1-q21.2

**Fig. 2:**
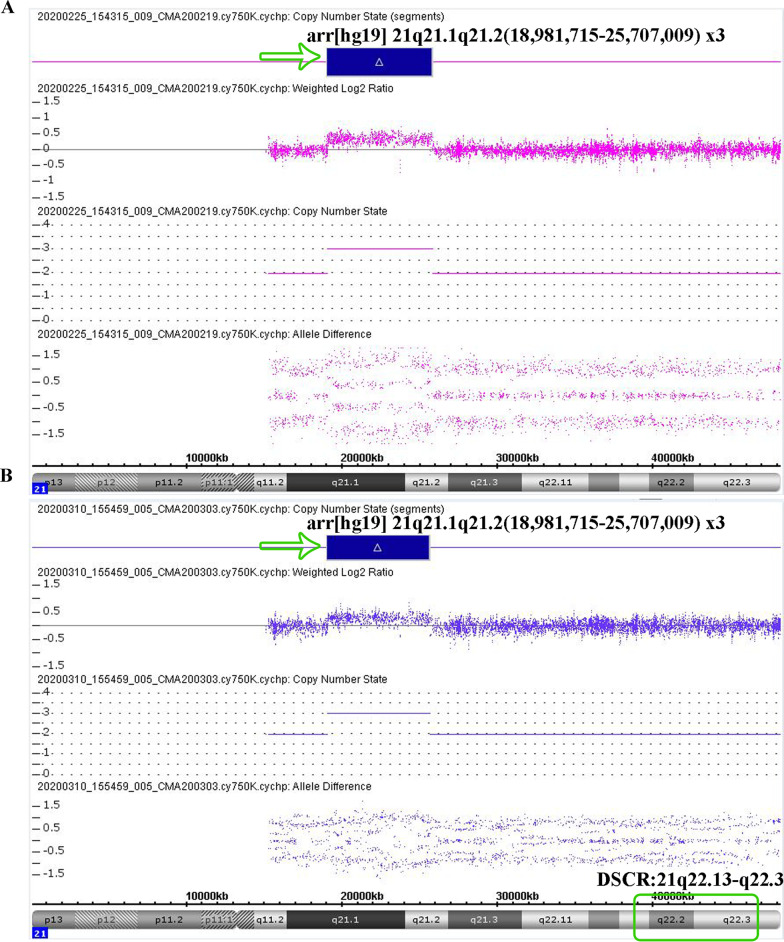
Chromosomal microarray analysis showed a 6.7 Mb duplication of 21q21.1-q21.2 for the fetus (**a**) and the pregnant woman (**b**)

The pregnant woman in this case was 27 years old, and this was her first pregnancy. All prenatal examination results were normal. Because serological screening was not performed at the appropriate gestational age, NIPT analysis was performed at 22 weeks of gestation (BGISEQ-500 SE35, 6 M reads). The NIPT results indicated a 5.8 Mb maternally inherited duplication of 21q21.1-q21.2 (Fig. 1). As a result, amniocentesis was performed at 27^+3^ weeks of gestation to evaluate the existence of this partial duplication in the fetus. The result of CMA was arr[hg19] 21q21.1q21.2(18,981,715–25,707,009) × 3, indicating a 6.7 Mb duplication of 21q21.1-q21.2 in the male fetus (Fig. 2a) (Affymetrix Cytoscan 750 K). CMA analysis of the pregnant woman at 31^+1^ weeks of gestation verified that this partial duplication of 21q21.1-q21.2 was indeed maternally inherited (Fig. 2b). The pregnant woman herself has an obviously normal phenotype, without any clinical abnormalities, as based on her medical history and physical examination results. Her prenatal examination results were also normal, except for the discovery of the maternally inherited partial duplication 21q21.1-q21.2. After genetic counseling, she and her family decided to continue the pregnancy. The Apgar score of the child at birth was good, and there was no abnormality at the 6-month follow-up.

## Discussion and conclusion

There are 20 genes located in the 21q21.1-q21.2 segment, including 4 OMIM (Online Mendelian Inheritance in Man) genes (*BTG3* (605,674), *CHODL* (607,247), *TMPRSS15* (606,635), and *NCAM2* (602,040)), according to Chromosome Analysis Suite (ChAS) of the Affymetrix Cytoscan 750 K microarray. A study in mice has revealed that *BTG3* expression in high in the ventricular zone of the developing central nervous system [9]. *CHODL* encodes a type I transmembrane protein homologous to C-type lectins that is mainly expressed in the vascular muscle of the testis, smooth muscle of the prostate stroma, heart muscle, skeletal muscle, crypts of the small intestine, and red pulp of the spleen [10]. The *TMPRSS15* gene encodes an enzyme that converts the pancreatic proenzyme trypsinogen to trypsin, which in turn activates other proenzymes, including chymotrypsinogen and procarboxypeptidases. Mutations in *TMPRSS15* cause enterokinase deficiency, a malabsorption disorder characterized by diarrhea and failure to thrive [11]. The *NCAM2* gene encodes a human neural cell adhesion molecule and is recognized as a candidate for involvement in some DS phenotypes. Nevertheless, the role of *NCAM2* in the pathophysiology of DS is unknown [12]. In summary, based on the current understanding of DS research, no OMIM gene in the 21q21.1-q21.2 segment was found to be definitively related to certain DS phenotypes.

To explore the pathogenic mechanism of DS, identification of genotype–phenotype correlations by studying rare cases of partial duplication chromosome 21 with or without the DS phenotype is extremely important. As more cases were reported, the term DSCR, a DS critical region, was first proposed by Rahmani et al. [13]. Among studies reported thus far, DSCRs that can lead to clinical phenotypes mainly include 21q22.3, 21q22.13, and 21q11.2-q21.1, among others [6, 14]. It has been believed that there are critical regions contributing to particular phenotypes but not for the majority of DS phenotypes [4–6]. Moreover, a highly restricted DSCR (HR-DSCR) of only 34 kb has been identified on distal 21q22.13 as the minimal region for which duplication is shared by all DS subjects and is absent in all non-DS subjects; this region contains no known gene and shows relevant homology only to the chimpanzee genome [6]. Phenotypes caused by DSCR include hypotonia, brachycephaly, sandal gaps, joint hyperlaxity, developmental delay, speech delay, congenital heart defects, etc. [8]. The case in this study carried a duplication of 21q21.1-q21.2 that did not involve DSCR of 21q22.13-q22.3 and presented no DS phenotype or any clinical manifestation. Most studies to date have focused on the identification of the pathogenic regions or genes of chromosome 21 associated with the DS phenotype [5]. Conversely, there have been nearly no studies targeting the nonpathogenic regions of duplication or partial duplication of chromosome 21, yet studies about nonpathogenic regions of chromosome 21 are as important as the identification of pathogenic regions for prenatal diagnosis.

In conclusion, our case clearly indicates that 21q21.1-q21.2 duplication is not included in the DSCR and most likely has no clinical consequences on phenotype. This case is not a direct confirmation of the pathogenicity of the DSCR but excludes the pathogenicity of duplication involving 21q21.1-q21.2. In addition, this case provides evidence to support cutting-edge analysis of partial duplication 21 with a normal phenotype and highlights that CMA can contribute to more accurate and efficient clinical diagnosis.

## Data Availability

The datasets generated during the current study are available in Gene Expression Omnibus (GEO) repository (accession number: GSE176138), direct link: https://www.ncbi.nlm.nih.gov/geo/query/acc.cgi?acc=GSE176138.

## Abbreviations

DSCR: Down syndrome critical region
NIPT: Noninvasive prenatal testing
CMA: Chromosomal microarray analysis
